# Severe hypertriglyceridaemia during treatment with capecitabine

**DOI:** 10.1038/bjc.2011.52

**Published:** 2011-03-08

**Authors:** L Javot, D Spaëth, J Scala-Bertola, N Gambier, N Petitpain, P Gillet

**Affiliations:** 1 Pharmacovigilance Center – Department of Clinical Pharmacology and Toxicology – University Hospital of Nancy, 29, avenue du Maréchal de Lattre de Tassigny – CO 60034 – 54 035 Nancy Cedex, France; 2 Department of Oncology, Clinic of Gentilly, 2 rue Marie Marvingt – 54 100 Nancy, France; 3 Department of Clinical Pharmacology and Toxicology – University Hospital of Nancy, 29, avenue du Maréchal de Lattre de Tassigny – CO 60034 – 54 035 Nancy Cedex, France

**Sir,**

We read with great interest the study of Michie *et al* (2010) evaluating capecitabine-induced hypertriglyceridaemia (CIHT). The authors suggest that it should be classified as a common adverse effect and we fully agree with their recommendation for targeted screening in patients with diabetes or pre-existing hyperlipidaemia. Here we report an additional case of severe CIHT in a patient with pre-existing hyperlipidaemia to focus attention on possible mechanisms.

A 50-year-old woman with a history of breast cancer, treated by surgery and hyperlipidaemia stabilised with pravastatin, was diagnosed in January 2009 with a progression of her breast cancer. Before receiving carboplatin, TAXOL (Bristol Myers Squibb, France) and bevacizumab, cholesterolaemia level was 6.68 mmol l^−1^ (2.59 g l^−1^) and triglyceridemia level was 2.51 mmol l^−1^ (2.20 g l^−1^) (Figure 1). During the 6-month treatment, a transient increase in triglycerides was observed and pravastatin was replaced by rosuvastatin. In July 2009, bevacizumab alone was administered once a week as maintenance treatment. In August 2009, because of secondary gastric and ganglion infiltration, carboplatin and taxol were stopped and the patient received a combination of capecitabine and bevacizumab. Nine cycles of this treatment were scheduled. After six cycles, in December 2009, highly raised triglycerides (26.77 mmol l^−1^ (23.48 g l^−1^)) and slightly raised cholesterol levels (8.75 mmol l^−1^ (3.39 g l^−1^)) were observed, without any clinical sign of pancreatitis. At no time had the patient reported alcohol consumption or dietary changes and no weight gain was observed. The replacement of rosuvastatin by fenofibrate was followed 1 week later by a significant decrease in her triglyceride level to 6.26 mmol l^−1^ (5.49 g l^−1^). However, 1 month later, the triglycerides level again increased to 11.33 g l^−1^ (9.94 mmol l^−1^). Owing to both side effects and progression of her cancer, capecitabine and bevacizumab were stopped and replaced by fulvestrant (FASLODEX; Astrazeneca, Cheshire, UK). A return to baseline triglyceridaemia values (3.27 mmol l^−1^ (2.87 g l^−1^)) was observed within 2 months.

Our case fulfils the criteria for a probable case of CIHT. Concomitant bevacizumab was not suspected because the increase in triglycerides during first-line chemotherapy resolved spontaneously, whereas treatment with rosuvastatin was unchanged.

We would like to propose two ways to improve our understanding of CIHT:

A defect of lipoprotein lipase (LPL) is linked with an accumulation of chylomicrons and/or of very low-density lipoprotein (VLDL). Kurt *et al* (2006) suggest that CIHT may appear more readily in individuals with hereditary LPL deficiency. It is worth noting that LPL defect also occurs in the case of an increase in its inhibitor (apolipoprotein CIII) and/or a decrease in its activator (apolipoprotein CII). As far as we know, the link between capecitabine and apolipoprotein CII/CIII levels has never been explored. We suggest that laboratory test of patient should include measure of LPL activity as well as of apolipoprotein CII/CIII levels.
As a triple prodrug, capecitabine is designed to be sequentially activated to 5-FU: capecitabine is hydrolysed in the liver to 5′-deoxy-5-fluorocytidine, which is then converted into 5′-deoxy-5-fluorouridine (5′-dFUR) and subsequently into 5-FU by thymidine phosphorylase. Many cases of severe hypertriglyceridaemia were described with capecitabine, but to the best of our knowledge only one study has dealt with the influence of 5-FU on serum and it did not report any changes in triglyceride level in human (Stathopoulos *et al*, 1995). Recently, a case of recurrent grade-4 hypertriglyceridaemia in a patient treated with capecitabine and tegafur/uracil was reported (Yildiz *et al*, 2010). This observation of hypertriglyceridaemia with another prodrug of 5-FU suggest that capecitabine and tegafur might share a mechanism responsible for hypertriglyceridaemia. Tegafur is bioactivated in 5-FU by thymidine phosphorylase in a single step. Considering that this enzyme is also responsible for the phosphorylation of 5′-DFUR in 5-FU, we suggest that thymidine phosphorylase could have a role in the generation of hypertriglyceridaemia. Thymidine kinase and thymidine phosphorylase compete for thymidine, and catalyse opposing synthetic and catabolic reactions implicated in proliferation and angiogenesis, respectively (Brockenbrough *et al*, 2009). As a modification of the expression and activity of thymidine kinase as well as a strong increase in the secretory phospholipase-A2 was observed in resistant colon cancer cells (Temmink *et al*, 2010), a link between thymidine metabolic pathways and phospholipids metabolism that may at least partially explain CIHT.

A lipid survey should be mandatory before initiation of capecitabine or other 5-FU prodrugs. Patients with dyslipidaemia should be closely monitored and further studied.

## Figures and Tables

**Figure 1:**
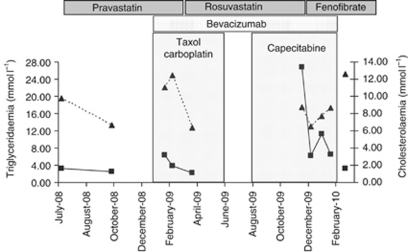
Clinical course of triglyceride (▪) and cholesterol (▴) levels in patients, expressed in mmol l^−1^.
